# A Systematic Review Evaluating the Impact of Fibre Supplementation on Gut Health and Other Clinical Outcomes in Adults with Haematological Malignancies During Haematopoietic Stem Cell Transplantation

**DOI:** 10.3390/nu17182973

**Published:** 2025-09-16

**Authors:** Fiona McCullough, Janice Cheung, Laura J. Miller

**Affiliations:** 1 School of Biosciences, University of Nottingham, Nottingham LE12 5RD, UK; 2 Department of Dietetics and Nutrition, Nottingham University Hospitals NHS Trust, Nottingham NG5 1PB, UK; 3 Centre for Rehabilitation and Ageing, School of Medicine, University of Nottingham, Nottingham NG7 2QL, UK

**Keywords:** gut health, dietary fibre, nutritional management, haematological malignancies

## Abstract

**Background:** Gut health is often disrupted in adults with haematological malignancies (HMs) receiving chemotherapy and haematopoietic stem cell transplantation (HCT). Microbial diversity is reduced, and both infection risk and inflammation increased. The role of dietary fibre in enhancing gut health, immune regulation, reducing complications, and improving clinical outcomes for people with HMs shows promise but the extent of their role remains unclear. **Objectives:** This systematic review evaluated the role of dietary fibre supplementation in adults with HMs undergoing HCT on gut health, immune function, and gastrointestinal health. This included assessment of differences between fibre types. **Methods:** A systematic search of PubMed and EMBASE was conducted following PRISMA guidelines, independently by two reviewers. Study quality was assessed using the Newcastle–Ottawa scale (NOS). **Results:** Of the 5023 studies after de-duplication, 63 remained after abstract and title screening, 59 studies were full-text screened, 56 studies were excluded due to language (*n* = 6), wrong intervention (*n* = 25), wrong population (*n* = 4), or reporting on unrelated outcomes (*n* = 21), and 3 studies met all inclusion criteria. Interventions included fructooligosaccharides (FOS), resistant starch (RS), and a glutamine, fibre, and oligosaccharide (GFO) prebiotic blend. Despite heterogeneity in measured outcomes, positive impacts on gut health, immune function, and gastrointestinal health were shown. **Conclusions:** Fibre supplementation represents a promising adjunctive strategy to improve clinical outcomes in adults with HMs undergoing HCT, by improving microbial diversity, increasing short-chain fatty acid (SCFA) production, and reducing incidence of acute GVHD. Further research is needed to establish specific recommendations for fibre in the nutritional management of patients with HM.

## 1. Introduction

High-fibre diets have been associated with reduced cancer risk in a range of cancers with differing levels of effect [1]. Fibre has also been associated with reduced bacterial dysbiosis and improved health outcomes in people with cancer, including blood cancers [2].

Haematological malignancies (HMs), including leukaemia, lymphoma, and multiple myeloma, are a group of cancers that significantly impact the blood, bone marrow, and lymphatic system [3]. HMs are a heterogeneous group of neoplasms, which occur when haematopoietic and lymphoid cells grow and divide uncontrollably during different stages of the differentiation process [3].

Haematopoietic stem cell transplantation (HCT) is a curative treatment for some HMs. Prior to HCT, patients will have received multiple rounds of chemotherapy to induce and maintain disease remission. Further conditioning chemotherapy is then given to clear blood and marrow stem cells. Stem cells are then collected from the patient’s own cells (autologous transplant) or a donor (allogeneic transplant) and transfused into the recipient. This results in naive blood cells and prolonged neutropenia with elevated infection risk [4].

While chemotherapy destroys the cancer cells, it also damages healthy cells like the colonocytes within the large bowel. This can lead to chemotherapy-induced gut dysbiosis, decreased beneficial bacterial diversity, and an increase in potentially harmful bacteria [5,6]. Use of prophylactic antibiotics to combat elevated infection risk during periods of neutropenia exacerbates dysbiosis [7] and reduces microbial diversity. Reduced microbial diversity has been linked to increased infection risk [6], poorer clinical outcomes, and reduced treatment efficacy [8].

Treatment-related complications, such as mucositis, graft versus host disease (GvHD), and fatigue, significantly affect patient quality of life [9]. Certain fibres have been shown to support gastrointestinal health and reduce treatment-related complications; however, maintenance of a diverse diet, high in fibre, can be difficult. Anorexia, treatment-related side effects, disease-related malnutrition, and fatigue can contribute to reduced dietary intake [10]. Guidelines on optimal approaches to support fibre intake and dietary education in this population are lacking and focus predominantly on energy and protein provision [11] over fibre and nutrient intake [12].

### 1.1. Graft vs. Host Disease

Graft versus host disease (GvHD), one of the main causes of morbidity and mortality in HCT recipients, has been linked to dysbiosis [13]. Emerging evidence suggests that gut microbiota composition and diversity may influence GvHD severity. Higher levels of fibre-loving butyrate-producing bacteria are also associated with lower incidences of acute GvHD [14].

GvHD is caused by immunocompetent donor T-lymphocytes identifying the recipient’s tissues as foreign due to histocompatibility mismatches and triggering an immune response. Manifestations in the first 100 days post-transplant are called acute GvHD (aGvHD) and predominantly affect the skin, gastrointestinal tract, liver, and lungs [3,13]. Chronic GvHD occurs after 100 days, affects any organ, and lasts for up to 7 years [15]. Risk of GvHD increases with donor and recipient age, human-leukocyte antigen (HLA) mismatch, conditioning regimen, and whether T-cells are depleted from donor cells [3,15].

aGvHD can affect up to 70% [16,17] of recipients but mostly at lower grades (1–2), which can be managed well with prophylaxis [3]. More severe aGvHD, grades 3–5, affects up to 15% of patients and is associated with irreversible deficits or death [16]. Overall survival from aGvHD has improved over time [17,18] due to prophylactic immunosuppressives, such as Calcineurin inhibitors (Tacrolimus or Ciclosporin), Methotrexate or Mycophenolate Mofetil (MMF), and high-dose cyclophosphamide, early post-transplant [17], alongside improved novel aGvHD therapies, such as Ruxolitinab (JAK-2 inhibitors) and Itacitinib (JAK-1 inhibitor). However, higher grades (III–IV) continue to be associated with worse morbidity and mortality in the short and long term (late effects) [16]. Some of the late effects include increased comorbidities, as well as physical and psychological stressors [16]. Symptoms vary depending on severity and impact on organs but include malabsorption, malnutrition, extensive skin rashes, and liver dysfunction.

### 1.2. Role of Dietary Fibre

Dietary fibre helps to maintain a healthy digestive system by adding bulk and moisture to stools, aiding peristalsis, and reducing constipation [19]. Certain fibres, known as prebiotics, are selectively fermented in the colon by gut microbes [20]. Fermentation by anaerobic bacteria produces short-chain fatty acids (SCFAs), such as acetate, propionate, and butyrate. These SCFAs support the survival and growth of a variety of gut bacteria, increasing microbial diversity [21]. They also regulate immune function by interacting with G-coupled receptors and promoting regulatory T-cells (Tregs) activity, dendritic cell function [2], and lowering pro-inflammatory cytokines [22]. SCFAs, especially butyrate, strengthen tight junctions between intestinal cells, supporting gastrointestinal integrity and reducing bacterial translocation and infection risk [23]. Fibre also confers other health benefits, such as the modulation of glucose and lipid metabolism, immune cell maturation, inflammation, appetite, and gastrointestinal health [20,21,24].

In people with HMs, fibre supplementation may enhance microbial diversity and SCFA production, supporting immunological function, preventing pathogenic bacterial overgrowth, and reducing oxidative stress [2].

### 1.3. Types of Fibres

Dietary fibre is present naturally in plant-based foods, such as cereals, vegetables, fruits, and nuts [25]. Fibre solubility and structure contribute to feelings of satiety, gut transit time, and insulin secretion, modifying glycaemic and lipid homoeostasis [26,27]. It also acts as an energy source for microbial fermentation [28].

Prebiotic fibres are commonly defined as a selectively fermented ingredient that results in health benefits for the host, via specific changes in the composition and/or activity of the gastrointestinal microbiota [29]. Specifically, prebiotic fibres should (1) resist digestion in the stomach (acidic pH and enzymatic hydrolysis) and absorption by the gastrointestinal tract and (2) support host health through selective stimulation of the growth and/or activity of intestinal (colonic) bacteria [29].

Most prebiotic fibres are oligosaccharide carbohydrates. They are classified by the degree of polymerisation, chain length, glycosidic links, and monosaccharide composition (glucose, fructose, galactose, or xylose) [29]. Their composition determines selective bacterial fermentation, metabolite synthesis, and associated health benefits or side effects, such as flatus [29,30]. Commonly accepted prebiotic fibres include Inulin-type fructans (Inulin and fructooligosaccharides (FOS)), galacto-oligosaccharides (GOS) [30,31], resistant starch (RS), and pectic oligosaccharides (POS) derivatives of pectin [31].

Fermentation of prebiotic fibres, such as RS and FOS, increases butyrate-producing bacteria and associated SCFA in the gut [30]. Prebiotics have been shown to alleviate mucosal injury, mucositis, and diarrhoea complications in people with cancer [32,33].

Higher dietary fibre intake has been linked to improved health and survival rates in people with cancer [34]. However, oral fibre intakes may need to be augmented by commercial supplements due to treatment-related side effects [25,27]. These commercial supplements can be single fibres or prebiotic blends [25].

This systematic review aims to evaluate the potential benefits of dietary fibre supplementation on the clinical outcomes of adults with HMs undergoing HCT, specifically the types and dose of fibre and their delivery methods, to inform recommendations for clinical practice where appropriate.

## 2. Methodology

### 2.1. Eligibility Criteria

This systematic review investigated the effects of fibre supplementation on the clinical outcomes of adults (over 18 years) with histologically confirmed HM receiving stem cell transplantation. HCT is a unique immunological infarct and thus studies of other cancers or treatments were excluded to reduce heterogeneity and to support interpretation of findings for clinical application. Paediatric populations were excluded due to immunological naivety and differences in microbiota between adults and children [35].

Orally administered fibre interventions considering dietary approaches to increase fibre intake, e.g., dietary education, supplementation (fortified nutritional supplement drinks with fibre), and therapeutic fibre provision, e.g., prebiotics, were included. Studies of artificial tube feeding were excluded. Intervention studies of synbiotic preparations (prebiotic and probiotic mixtures) were also excluded due to uncertainties regarding the safety of probiotics in this population.

The search was not limited by study type, as a range of designs were expected from feasibility studies to randomised control trials (RCTs). No restrictions were made by outcome. While the review primarily sought to identify clinically relevant measures for trials of fibre interventions within adults receiving HCT, secondary outcomes were also reported to understand delivery issues, e.g., adherence, and theoretical mechanisms of action, e.g., microbiome and immunological markers. A summary of the inclusion criteria is available in Table 1.

### 2.2. Literature Search Methodology

The systematic review followed the PRISMA (2020) guidelines for transparency when reporting all stages of the search process [36]. The search process included three stages: identification, screening, and inclusion. Identification: Key terms and their associated derivatives were mapped to a Medical Subject Heading (MeSH). A template strategy was developed in PubMed and adapted for EMBASE using relevant Boolean operators, wild cards, and dictionary terms. This refined search strategy was then used to conduct a bibliographic search within PubMed and EMBASE, as follows: (“H#ematological malignanc*” OR “H#matological cancer*”) AND (“Fibre supplementation”) AND (“Clinical outcome*” OR “Gastrointestinal side effects” OR “Quality of life”). The search was conducted in October 2024. Reference lists of included studies were screened for additional articles. No restrictions were placed on the time of publication, so a broader range of studies and evidence could be included, due to limited research in this area. Studies not published in English were excluded due to no accessible translation resources.

Screening: Duplicates were removed and titles and abstracts were screened against inclusion criteria (Table 1) by J.C., and any queries were checked by F.M. Full papers were then reassessed against inclusion criteria to identify final papers for analysis.

#### Data Extraction

**Figure 1:**
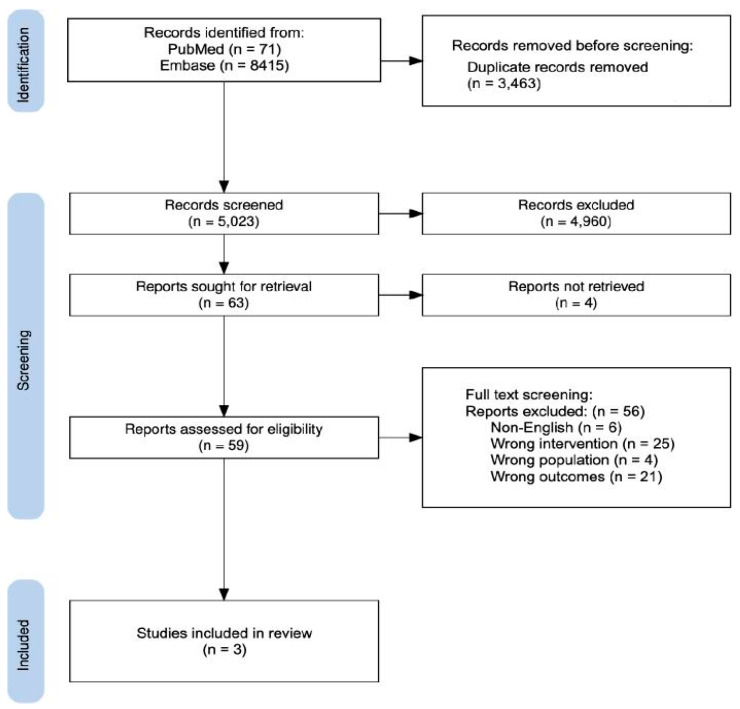
PRISMA flow diagram detailing the search process and the resulting studies identified [24].

Search results were downloaded, and a data extraction table was developed. Search results and decision-making were reported in a PRISMA flowchart (Figure 1). Data on study design, population, intervention (timing, constituents, and dose), and outcomes were extracted. Frequency counts, percentages, averages, and *p*-values were reported where relevant. Results are reported narratively, as a statistical meta-analysis was unable to be conducted due to the high heterogeneity of the selected studies.

### 2.3. Quality Assessment

All identified studies were non-randomised and, as such, the Newcastle–Ottawa scale (NOS) [37] was used to assess the quality of the included studies [38].

The NOS rates the studies based on the selection of study groups, compatibility of the groups, and assessment of the outcomes or exposures. A star is given for each of these aspects, if the study meets predefined criteria. In total, a maximum of nine stars can be awarded to a study as an indication of its quality. Studies scoring 7 or higher are considered high quality with a low risk of bias [38].

## 3. Results

### 3.1. Study Selection

Of the 5023 de-duplicated records, 4960 were excluded after title and abstract screening against inclusion criteria. Of the 63 papers identified for article retrieval, 4 were not accessible, leaving 59 studies for full-text review. Following full-text screening, three studies were identified that met the inclusion criteria (Figure 1).

### 3.2. Quality Assessment Outcomes

The results of the quality assessment analysis (Table 2) showed the included studies to be of high quality with a low risk of bias.

### 3.3. Study Design

No randomised trials were identified—study designs included a prospective cohort study [39], a prospective study [40], and a non-RCT [41]. Two out of three studies used historical control groups as comparators [40,41], while the other study used a standard control group [39].

### 3.4. Population Demographics and Treatments

Here, 3 studies of 250 participants were included, and all received allogeneic stem cell transplant and were heterogenous for HM. The majority of HMs were acute myeloid leukaemia across all studies. Conditioning regimens varied between studies, from reduced intensity conditioning (RIC) only [39] to mixed cohorts of high and RIC regimens [40,41]. All recipients received some form of GvHD prophylaxis of either a calcineurin inhibitor, e.g., Tacrolimus (FK506) with Methotrexate [39,40,41], Cyclosporine alone [40], or with Methotrexate [41]. All had some form of prophylactic antibiotic provision. Participants had an average age of 47–65 years. Age ranges were grouped as < or ≥55 years in two studies, where one had nearly 50% more under 55 years [40], compared to nearly three-quarters over 55 years in the other [41]. All studies had more males than females. Ethnicity was only reported in one study [39]. See the Supplementary Materials S2 for further potential confounder details.

### 3.5. Intervention Types

Each study used a different formulation of prebiotic fibre(s), timing, and method of administration. One study used a Chicory-based FOS [39], and two used a commercial prebiotic mix [40,41]. Overall, supplementation was generally well tolerated.

The chicory-based FOS supplement, Fibrulose F97 (Cosucra, Warcoing, Belgium), was used to assess tolerability and acceptability during HCT [39]. Participants were divided into three groups receiving different doses of FOS (5 g, 10 g, or 15 g per day), which could be either dissolved in a drink or added to food. Supplementation started the day of hospital admission (5 days pre-transplantation) for a total of 21 days. The control group did not receive FOS (*n* = 15 intervention vs. *n* = 16 control) [39].

Another study used a combination of amylofibre SH, a cornstarch containing 70% resistant starch, and a prebiotic mixture of glutamine, fibre, and oligosaccharides (GFO) [40]. This intervention was administered at the beginning of pre-transplant conditioning until 28 days post-transplantation. Participants received 8 g of RS at lunch and dinner, and 1 pack of GFO (3 g of glutamine, 5 g of polydextrose, and 1.45 g of lactosucrose) at breakfast daily. This was compared against a historical control (*n* = 49 intervention vs. *n* = 142 control) [40].

The final study used a higher dose of the same GFO supplement (Otsuka Pharmaceutical Co., Ltd. (Tokushima, Japan)) without resistant starch and compared this to a non-interventional usual care control group (*n* = 22 intervention vs. *n* = 22 historical control) [41]. Two packs of GFO (72 kcal, 6 g of glutamine, 10 g of dietary fibre (polydextrose), 3 g of oligosaccharide (Lactosucrose), and 2.4 mg of sodium) were dissolved in 200 mL of water and administered orally three times per day. The intervention began 7 days before starting pre-transplant conditioning regimens and continued until 28 days post-transplantation. The intervention was stopped if vomiting occurred [41]. All participants (both arms) received a probiotic lactobacillus preparation (Biofermin-R, Biofermin Pharmaceutical Co., Ltd., Kobe, Japan). Table 3 provides an intervention summary.

### 3.6. Study Outcomes Measured

A variety of outcomes were measured across the studies to assess the impact of fibre supplementation and prebiotics on HCT recipients. The results were considered statistically significant at *p* < 0.05 [42].

Table 4 provides a summary of the main clinical and microbiome outcomes.

### 3.7. Gastrointestinal Outcomes

Table 4 summarises the impact of fibre supplementation on gastrointestinal outcomes. Diarrhoea and mucositis (oral and gastrointestinal) are common complications during HCT treatment [43]. The duration and severity of diarrhoea were significantly reduced in the intervention groups in two studies, relative to their respective control groups [40,41]. In the RS + GFO group, diarrhoea lasted for a median of seven days, compared to nine days in the historical control group (*p* < 0.05). Additionally, a greater percentage of patients in that group experienced no diarrhoea (17% vs. 7%, *p* < 0.05) [40]. Similarly, in the GFO group, patients had significantly fewer days with severe diarrhoea (grade > 3: 0.86 days vs. 3.27 days, *p* = 0.001) and moderate diarrhoea (grade > 2: 3.73 days vs. 7.68 days, *p* = 0.0001), although there were no significant differences in the maximum diarrhoea grade between the groups (grade 2.00 vs. 2.68, *p* = 0.68) [41].

The duration and severity of mucositis were also measured. In the RS + GFO group [40] and GFO only group [41], patients experienced reduced durations of mucositis compared to their respective control groups. In the RS + GFO group, the duration of moderate-to-severe-grade oral mucositis was 11 days, compared to 14 days in the control group (*p* < 0.05) [40]. The GFO group experienced fewer days with severe mucositis (grade > 3) of 3.86 days compared to 6 days for patients in the control group (*p* = 0.033). There were, however, no significant differences in the maximum mucositis grade between the GFO and control groups (grade 1.55 vs. 2.05, *p* = 0.20) [41].

**Table 3:** A summary of prebiotic fibre interventions and outcomes within included studies.

Author, Year, Country	Study Design	Patient Population and Sample Size	Intervention	Outcomes Measured	Results Summary
**Andermann et al., 2021 [39]**	Prospective cohort study	Adults with HM Reduced-intensity conditioning Allogeneic HCT I: *n* = 5 C: *n* = 6	**Intervention:** Single prebiotic (FOS) **Daily dose:** Prebiotic FOS (Fibrose F97, Cosucra, Belgium) 5, 10, or 15 g (cohort divided into 3 groups of *n* = 5) **Start:** Day of hospital admission; 5 days prior to transplant **Stop:** 21 days post-transplantation	**Primary:** FOS tolerability, adverse events, feasibility of delivery **Secondary:** aGvHD incidence (CTCAE v4.0), survival, *Clostridioides difficile*-associated diarrhoea (CDAD) incidence, Shannon diversity, key taxa changes, SCFA levels, FOXP3 + Treg concentrations, and CTLA4+ T-cells	Feasibility met the preset goal. Faecal butyrate levels were significantly higher in the intervention group, plus the dominant plasma metabolites were significant more stable compared to controls. Also, significant alterations in intestinal and plasma metabolites versus control.
**Yoshifuji et al., 2020** **Japan [40]**	Prospective study	Adults with HM Reduced- and high-intensity conditioning Allogeneic HCT I: *n* = 49 C (historical): *n* = 142 Faecal samples collected: I: *n* = 30, C: *n* = 72	**Intervention:** Prebiotic **Daily dose:** 16 g resistant starch (RS) and 1 pack GFO (prebiotic mixture) RS: 8 g of Amylofibre SH (J-Oil Mills Inc, Tokyo, Japan) 2 × per day at lunch and dinner 1 pack of GFO (Otsuka Pharmaceutical Factory Inc, Japan) at breakfast—3 g glutamine, 5 g polydextrose, 1.45 g lactosucrose **Start:** Pre-transplant conditioning regimen **Stop:** Day 28 post-transplantation **Control:** Usual care	**Primary:** Incidence and duration of diarrhoea (CTCAE v4.0) and oral mucositis (OAG score) **Secondary:** Incidence of aGvHD and TPN use **Additional measures:** Prebiotic intake, fibre provision, opioid use, survival, Shannon diversity, butyrate-producing bacteria abundance, and butyrate concentration	Prebiotic intake mitigated mucosal injury and reduced the incidence of all aGVHD grades. Intervention group had significant maintenance of gut microbial diversity and preserved butyrate-producing bacterial population. Also, post-transplantation faecal butyrate concentration was maintained or increased more frequently than in the control group.
**Iyama et al., 2014** **Japan [41]**	Non-RCT	Adults with HM Reduced- and high-intensity conditioning Allogeneic HCT I: *n* = 22 C: (historical): *n* = 22 Matched-pair control group Controls were matched to cases at a 1:1 ratio based on factors like age, disease status, and pre-transplant conditioning to reduce bias	**Daily dose:** 2 packs of 15 g GFO 3 × per day (Otsuka Pharmaceuticals Co., Ltd.) Per pack dose: 36 kcal, 3 g glutamine, 5 g dietary fibre, 1.5 g oligosaccharide, and 1.2 mg sodium, dissolved in 200 mL of water **Start:** 7 days pre-transplant conditioning regimen **Stop:** 28 days post-transplantation. Early stop if vomiting occurred	**Primary:** Severity and incidence of diarrhoea and oral mucositis (CTCAE v4.0) **Secondary:** Days of diarrhoea, mucositis, weight loss, TPN, microbial infection, hospital days, survival, aGvHD incidence and severity, and relapse rate	Fewer days of diarrhoea grades 3–4 in patients receiving the intervention than in those who did not; also, days of mucositis grades 3–4 at day 100 was 100% in the GFO group and 77.3% in the control group. Weight loss and the number of days of intravenous hyperalimentation were better in the intervention group. Other outcomes were not affected.

This table provides a summary of key study and intervention characteristics, outcomes and reported findings of included studies. RCT—randomised controlled trial; HM—haematological malignancy; I—Intervention; C—control; HCT—haematopoietic stem cell transplantation, FOS—fructo-oligosaccharide; GOS—galacto-oligosaccharide; CTCAE—Common Terminology Criteria for Adverse Events; TPN—total parenteral nutrition; SCFA -Short chain fatty acids; aGvHD—acute graft versus host disease.

**Table 4:** Reported outcome measures within included studies.

Author (Year)	Group	Reported Clinical Outcomes											Reported Microbiome/Metabolome Outcomes		
		Wt.	Diarrhoea			Mucositis		Infections/Immunological Markers			aGvHD	Disease	Microbiome/Metabolome		
		Loss (Kg)	Duration (Days)	Incidence (%)	Max. Grade	Duration (Days)	Max Grade	Days of Fever >38.5 °C	Reported Infection	Immune Cell Markers	Incidence *n*, %	Survival Rate	SI	Butyrate Bacterial Abundance	Faecal Butyrate Levels
**Anderman et al (2021) [39]**	**FOS**								**CDAC** 0, 0% **BSI** 6.40%	**D28 CTLA4+ CD4+ T-Cells** higher FOS group *p* < 0.001 **D28 FOXP3 + CD4+ T-cells** higher FOS group *p* = 0.013	9, 60% Grades I–IV	Overall mortality 6 months, 2.13% 12 months, 3.20%	No significant difference between groups and recovered to baseline by D + 100		No significant differences between groups
	**Control**								**CDAC** 2.13% **BSI** 3.19%		9, 56% Grades I–IV	Overall mortality 6 months, 2.13% 12 months, 6.38%			
**Yoshifuji et al. (2020) [40]**	**RS + GFO**		Duration less in RS + GFO gp * (*p* = 0.049) RS + GFO, 7 days; Control, 9 days	Incidence of no diarrhoea RS + FOS, 17%; Control, 7%	Grade 3+ similar between groups	Moderate–severe oral mucositis less in FOS group * (*p* < 0.001) 11 days vs. 14 days	MAX OAG higher in control than prebiotic (*p* = 0.101)				Cumulative incidence is lower in RS + GFO group at D100 for **All grade aGvHD** 53.1% vs. 73.2% (*p* = 0.004) **Grades II–IV** 24.5% vs. 46.1% (*p* = 0.006) **Skin—all grades** 44.9% vs. 63.4% (*p* = 0.010)	OS higher in RS + GFO group No significant difference in NRM	SI higher pre-HCT in control * (*p* = 0.011), D28 no significant difference in SI between groups (*p* = 0.444)	Butyrate-producing bacterial levels sustained in RS + GFO group from pre-HCT to D28 *p* = 0.013)	Butyrate levels higher pre-HCT in control *p* = 0.013)
	**Historical Control**														
**Iyama et al. (2014) [41]**	**G.FO**	Lower % wt. loss 2.15% GFO vs. 6.42% * *p* < 0.001	Less days grades 3–4 with GFO *, 0.86 vs. 3.27 days **(*p* = 0.001),** **or grade 2,** 3.73 vs. 7.68 days		No difference in max. grade score, 2 (GFO) vs. 2.68 (control) (*p* = 0.68)	Less days grades 3–4 with GFO, 3.86 vs. 6 days (*p* = 0.033)	No significant difference in max. grade score, 1.55 (GFO) vs. 2.05 (control) (*p* = 0.2)	No difference in days of fever, 0.73 days	No difference in documented microbial infections, 4/22 (GFO vs. 5/22 control		No significant difference in incidence and severity	OS greater in GFO group D + 100 (100% vs. 77.3%) * (*p* = 0.0091)			
	**Control**														

This table shows the key outcomes reported by included papers, where there is a significant difference between studies. *p*-values are reported and * assigned if *p* < 0.05. Other reported differences are nonsignificant. aGvHD—acute graft versus host disease; OS—overall survival; NRM—non-relapse mortality; CDAC—*Clostridium difficile*-associated colitis; BSI—blood stream infection, which consisted of organisms thought to have originated from oral or gastrointestinal microbiome, such as *Enterobacteriaceae*, *Streptococcus mitis*, and *Enterococcus* spp.; OAG—Eilers’ Oral Assessment Guide.

#### aGVHD Incidence and Risk

aGVHD is a common complication during HCT treatment [43]. Table 4 shows the incidence of aGVHD, which was measured across all three studies. The cumulative incidence of aGVHD by day 100 was significantly lower in the RS + GFO group compared to its historical control group (*p* < 0.05). The incidence of grades 2–4, more severe aGVHD, was also significantly reduced in that study (*p* < 0.05) [40]. However, in the other two studies, there were no significant differences in aGVHD incidence or severity between their intervention and respective control groups [39,41].

### 3.8. Nutritional Outcomes

Patients in the GFO only group experienced significantly less weight loss during the study period compared to the control group (2.15 kg vs. 6.42 kg, *p* < 0.001). This highlights the potential of GFO to help to preserve nutritional status during HCT [41]. One study also reported GFO supplementation association with a significant reduction (*p* = 0.001) in the days of total parenteral nutrition use [41].

Habitual dietary intake including fibre was not reported in any study. Proportional consumption of prebiotics and methods for calculation were reported in one study [40]. Overall, studies reported good tolerance of prebiotic interventions, but total dose consumption was limited by treatment-related side effects, e.g., nausea and mucositis. In one study, intake was limited by up to 50% [41]. Minor symptoms of flatulence and abdominal distention were found with FOS supplementation alongside a dosing threshold of 10 g [39].

### 3.9. Microbiological Outcomes

Microbiological outcomes, particularly gut microbiota composition and function, were also reviewed, where reported stool samples were analysed for microbial diversity using the Shannon diversity index (SI). The SI is a quantitative measure used to assess biodiversity, considering both the species richness (number of different species) and evenness (how equally abundant the species are). It is calculated using a formula, where the resulting value is between 0 and 1. A higher SI value indicates greater diversity [44]. Changes in the abundance of butyrate-producing bacteria were also assessed, and faecal concentrations of SCFAs were measured to evaluate microbial metabolic activity.

Two studies collected stool and blood samples for microbial and SCFA sampling [39,40]. These measured outcomes are critical for understanding the role of fibre in maintaining gut health during HCT [45]. Microbial biodiversity by the SI was maintained in the RS + GFO group [40] when compared to the historical control group (*p* < 0.05) during HCT. In the same study, the intervention group showed better maintenance of butyrate-producing bacteria compared to controls (*p* = 0.027), and faecal butyrate levels were also better maintained (*p* < 0.05) [40].

Conversely, the study using FOS showed no significant differences in microbial diversity by the SI or faecal butyrate levels compared to its control group [39]. However, the FOS group showed a nonsignificant increase in blood stream infections, including Enterbacteriaceae, Streptococcus mitis, and *Enterococcus* spp., but lower Clostridium incidence of difficile infection [39].

In both studies, Shannon diversity significantly declined post-HCT, mostly recovering back to baseline by day 100+ [39]. Andermann et al. also conducted multi-dimensional scaling and found that individual pre-transplant Shannon diversity was most predictive of a higher post-HCT diversity, over FOS supplementation.

Only one study reported individual taxa changes [39] during HCT, with the most significant changes in *Ruminococcus* spp., *Clostridium* spp., and *Enerbacteriaceae bacterium*. Neither study provided sufficient information to describe differences in species at any stage. Microbial diversity is affected by a range of confounders, including antibiotic use, dietary intake, age, and gender, and their reporting is available in the Supplementary Materials S2.

### 3.10. Immunity, Infection, and Fever

In one study, the GFO group experienced fewer days with fever (>38.5 °C) compared to controls (0.73 days vs. 1.41 days), though this difference was not statistically significant (*p* = 0.41) [41]. Microbiologically documented infections were reported in 4/22 patients in the GFO group and 5/22 in the control group (*p* = 0.71), showing no significant difference in infection rates, although the types of bacteria identified varied slightly [41].

CTLA4+ T-cells, a marker of CD4+ T-cell activation, linked with the induction of intestinal regulatory T-Cells (Tregs), support intestinal immunological homeostasis. Within the study of FOS supplementation, CTLA4+ T-cells were found to be significantly increased in the FOS group (*p* = 0.013), displaying improved immune function [39]. This response was not dose-dependent, with the same effect observed at 5 and 10 g FOS [39].

## 4. Discussion

In this systematic review, we sought to identify current literature on the role of fibre as a supportive therapy for adults with haematological malignancies receiving a stem cell transplant. While encouraging preliminary data showed prebiotic supplementation to be acceptable and feasible to deliver, with potential to modulate severity of mucositis, infections and aGvHD results were inconclusive, and further large-scale studies are needed to determine the efficacy, dose, and timing.

### 4.1. Fibre Structure and Composition May Explain Variations in Outcomes

Prebiotic fibre constituents and dosing were heterogenous (FOS, RS, and GFO), limiting meta-analysis. While no study was powered to measure efficacy, all showed potential benefits of prebiotic fibre supplementation with varying significance [39,40,41]. Significant differences in clinical outcomes (aGvHD and mucositis) were only seen with a prebiotic mixture of glutamine, fibre (polydextrose), and an oligosaccharide (GFO), lactosucrose, given alongside a resistant starch [40,41]. While FOS alone was not associated with significant differences in microbial diversity, SCFA levels, and aGVHD incidence. However evidence of immunological upregulation was indicated by significant changes in CTLA4+ T-cell levels [39]. These variations in outcomes between studies may in part be explained by the different fibre analogues and their selective bacterial fermentation [30,46].

Studies included either a fructooligosaccharide (FOS), chicory root (cFOS) [39], or galacto-oligosaccharide (GOS), lactosucrose [40,41]. Both differ in their monosaccharide composition, glycosidic linkages, and degree of polymerisation, which may influence health effects [47]. cFOS is normally a longer polymer consisting of fructose (60–80%) and glucose [48], whereas lactosucrose is a trisaccharide of galactose, glucose (from lactose), and fructose (from sucrose) [49]. These different monosaccharides, their chain lengths, and their linkages result in differentially selective fermentation by bacteria and altered rates of fermentation.

FOS has been found to promote *Bifidobacterium* at doses of 7.5–25 g/d for 4 weeks or more [47]. *Bifidebacterium* synthesises SCFAs, acetate, and lactacte [47] from FOS and is associated with health benefits, such as vitamin absorption, bone health, immunity, gut health, and the promotion of other beneficial flora [47]. FOS is additionally fermented by a broad diversity of butyrate-producing bacteria, e.g., *Faecalibacterium*, *Eubacterium*, and *Roseburia*, and low levels of *Bacteroides* [48]. Fructooligosaccharide fermentation increases intestinal lumen acidity and synthesis of SCFAs, e.g., butyrate, propionate, and acetate, in the caecum. These SCFAs downregulate pro-inflammatory cytokines and reduce lymphocyte activation and proliferation [47]. Beneficial increases in butyrate-producing bacteria are also associated with higher luminal butyrate levels, a primary energy source for colonocyte health [47,48]. In comparison, lactosucrose is not directly fermented by butyrate-producing bacteria; instead, the lactate and acetate synthesised during fermentation by *Bifidobacterium* and *Lactobacillus* [49,50] can act as a substrate for butyrate producers.

Interestingly, only supplementation with RS + GFO showed evidence of preservation of microbial diversity, butyrate producers, and butyrate levels [40], and not GFO [41] or FOS alone [39]. This may reflect the additive benefit of resistant starch or the lower relative total fibre provision and doses of FOS and GOS in these studies.

Resistant starch (RS) is the fraction of starch that is not hydrolysed in the small intestine and fermented in the colon [51]. It has been shown to improve glycaemic and lipid metabolism and SCFAs’ synthesis, including butyrate, propionate, and acetate [52]. However, fermentation can lead to bloating and discomfort, as observed in irritable bowel syndrome.

Glutamine acts as a fuel source for some immune cells, e.g., macrophages and lymphocytes [53], and may aid mucositis, T-cell proliferation, and suppression of inflammatory cytokines [54]. However, some leukaemias and lymphomas may be glutamine dependent and compete with immune cells for glutamine [55,56]. As such, further mechanistic understanding of relative glutamine uptake by immune and leukaemic cells during supplementation and the bioavailability in prebiotic mixtures is warranted. Despite this, in the two studies of GFO supplementation there were no observable increases in mortality following supplementation [40,41].

Polydextrose is a synthetic, non-digestible oligosaccharide. Its highly branched structure slows fermentation compared to other short-chain oligosaccharides, reducing and slowing gas production [57]. A daily intake of 4–12 g has been shown to be tolerated, and positive microbiotal changes have been established between 8 and 21 g per day [57]. Within the included studies, participants received 5 g [40] or 30 g [41], with no reported side effects at either dose. Studies of polydextrose supplementation have shown variable and inconsistent changes in microbiota profiles [58,59] alongside inconclusive changes in SCFAs, which may in part be explained by animal models that indicate this could be a result of increased colonocyte absorption rather than reduced synthesis [59,60]. While the evidence in humans of health benefits is limited for polydextrose, its ability to offset the gastrointestinal effects of other short-chain oligosaccharides may help with tolerance of mixed prebiotic fibres.

### 4.2. Changes in Microbiota Were Underreported and Limited by Pre-Transplant Dysbiosis

A diverse gut microbiome is increasingly recognised as crucial for overall health, particularly in immuno-compromised people [61]. Where reported, there was initial maintenance of microbial diversity and abundance of butyrate-producing bacteria with fibre supplementation [39,40]. However, overall abundance declined for all participants in the post-transplant period, recovering by day 100 [40]. Individual pre-transplant Shannon diversity was found to be most predictive of a higher post-HCT diversity, over FOS supplementation [40]. If prebiotic fibres provide a food source for bacterial fermentation, then low relative abundance of fibre-loving bacteria at the initiation of prebiotic interventions may limit their effects.

Feasibility trials of faecal microbiota transplantation (FMT) in HCT recipients to increase microbiota diversity and recovery have proven to be safe [62,63,64]. FMT involves donor stool transfer (capsules, enemas, nasojejunal, or gastric feeding tube) into the recipient patient’s gut [62]. FMT has been associated with improved microbial diversity, reduction in *Enterococcus*, associated with GvHD, and expansion of *Collinsella*—a butyrate-producing bacteria [62]. As such, stratification of patients by baseline diversity with low-diversity individuals offered FMT prior to or alongside prebiotic fibre may support greater benefits. However, data on the safety of FMT prior to engraftment are still needed.

Andermann et al. [39] reported significant changes in *Ruminococcus* spp., *Clostridium* spp., and *Enterobacteriaceae bacterium*. However, this is difficult to interpret, as different species within each genus may be commensal or opportunistic. Increases in *Enterobacteriaceae* are also often seen following antibiotic use, due to bacterial overgrowth when there is low relative abundance of other species.

The preservation of microbial diversity and SCFA-producing bacteria suggests that fibre supplementation interventions may support gut integrity [24], immune function, and inflammation reduction [65]. These are important factors in reducing the impact of inflammatory-mediated side effects, e.g., aGVHD, reducing opportunistic infections, and improving clinical outcomes [66].

### 4.3. The Role of Prebiotic Fibre Supplementation on aGvHD Remains Inconclusive

Changes in aGvHD incidence and severity (grades 2–4) were mixed, with improvements only observed with GFO plus resistant starch supplementation [39,40,41]. This variability may be attributed to differences in fibre types, dosages, and patient demographics—including conditioning, HLA status, age, antibiotic use, habitual diet, and microbiome prior to HCT.

Interestingly, prebiotic supplementation was specifically associated with a reduction in skin GvHD at all stages (*p* = 0.01) and higher grades > 2 (*p* = 0.006) [40], with an indication that prebiotic effects were augmented by a more diverse Shannon index at baseline [41]. The mechanisms by which prebiotic fibres may influence aGvHD are not fully elucidated, and no study provided a theoretical model for their interventions. However, all included studies proposed that prebiotics and their constituents potentially reduced inflammatory cascades and thus aGvHD severity. SCFAs synthesised by butyrate-producing bacteria are thought to downregulate IL-10 expression and inhibit NF-κB signalling and histone deacetylase (HDAC)-mediating anti-inflammatory effects [67]. Growing evidence suggests that intestinal microbiota are dysregulated in patients with aGvHD [68]. Higher microbial diversity has also been associated with lower risk and severity of GvHD.

### 4.4. Prebiotics Appear to Offer Differential Improvements in Mucositis

Incidence and severity of mucositis (oral and gastric) were measured in all studies. Fibre supplementation demonstrated significant benefits for gastrointestinal health in patients with HM undergoing HCT. The reduction in diarrhoea duration and incidence, as well as the decreased severity of mucositis, suggested that fibre may help maintain gut integrity during the intensive treatments [69]. This may reduce side effects and complications associated with the supportive therapies, potentially improving patient comfort and quality of life, and lowering healthcare costs [70]. Side effects, such as abdominal pain and bloating, have been reported in other settings [71,72].

### 4.5. Limitations

While the search strategy was comprehensive, it identified no additional prebiotic studies to a 2023 scoping review by Anderson et al. [8], but it did identify additional studies to a recent systematic review by Gardiner et al. in 2025 [73], potentially due to different methodologies.

One feasibility study of 20 g of resistant potato starch (Bobs red mill) supplementation in adults with HM (*n* = 10) receiving allogeneic HCT [74] was not identified by this search or previous searches [8,73]. The lack of identifiable prebiotic fibre terms in the title and specified use of the term resistant potato starch in the abstract may have led to early title exclusion. Incorporation of search operators, such as ‘resistant adj2 starch’, may mitigate against this.

Additionally, evidence showed that baseline SI abundance may limit the effects of prebiotics. The exclusion of symbiotic interventional studies (prebiotic + probiotic mix) potentially limits understanding of prebiotic potential in populations with low relative abundance prior to HCT. Yazadandoust et al., in 2023, conducted a symbiotic supplementation trial of Familact (Zist Takhmir, Tehran, Iran), a FOS prebiotic + 10^9^ CFU 7-mixed strain bacterial probiotic, in adults with HM receiving HCT [75]. The symbiont supplement was given once daily from day 21 to day 0 (day of transplant; *n* = 20), compared to a non-interventional control (*n* = 20), which reported less mucositis and recurrence of the disease in the intervention group [76]. The degree to which each constituent contributed to this change is difficult to interpret or compare against other studies, as the amount of fibre provided is not described. A summary of these additional studies [74,75] and their relevant outcomes is available in the Supplementary Materials S3a–c.

The heterogeneity of the study designs, fibre doses, types, and outcomes measured prevented a meta-analysis and led to limited interpretation. No study provided a theoretical model that explained the proposed mechanisms underlying the intervention. Patient and public involvement in study development was also not reported in any study. Sample sizes of each study were small, and none were powered to detect efficacy. Two of the three included studies used historical control groups, which are less reliable and could introduce biases due to potential changes in clinical practice or patient characteristics over time [76]. Despite the historic controls, no statistically significant differences in baseline demographics were reported.

As well as natural prebiotic sources, e.g., Jerusalem artichoke and chicory, synthetic prebiotics are incorporated in a range of foods as fat replacers in dairy and sweeteners in low-calorie foods [77]. However, no study reported habitual intake and thus the influence of habitual dietary intake on clinical and microbiota outcomes in these supplementary trials is lacking. Standard antibiotic regimens were reported in two studies, and the relative use of antibiotics with high anti-anaerobic activity were only reported in one. While this study showed similar use between controls and intervention groups [39], understanding of antibiotic use in other studies as a confounder could not be considered.

### 4.6. Implications for Clinical Practice

Fibre supplements were generally well tolerated; however, treatment-related side effects limited the capacity to achieve target dosing in most studies. Diets high in prebiotic fibres could reduce HCT-associated complications but are dependent on type, dose, and timing. Prebiotic fibre provision should be considered alongside comorbidities and clinical symptoms, e.g., stool output. Fibre provision may be contraindicated in gastrointestinal comorbidities, such as Crohn’s disease [78].

This suggests that fibre may be beneficial in adults with HM receiving HCT and should be considered as part of dietary advice. Microbial augmentation in those with lower baseline microbial abundance (SI) via a combined probiotic [75] through a synbiotic supplement or faecal transplantation may augment prebiotic benefits, but results vary, and further research is needed [18,75]. Prebiotic mixtures containing specific amino acids should be carefully considered aligned with HM aetiology and metabolism, due to potential augmentation of some cancer cells. As such, clinicians should consider fibre supplementation based on patient characteristics, treatment protocols, and specific health goals to provide better treatment plans.

## 5. Conclusions

Despite the inclusion of high-quality and relevant studies, significant heterogeneity in fibre dose and composition, alongside underpowered small samples, limited comparisons and definitive conclusions.

Overall, fibre interventions were generally well tolerated. A supplement of glutamine, fibre (polydextrose), and lactosucrose plus resistant starch showed the most promise, with significant improvements in aGvHD and mucositis incidence and severity. Low relative microbial abundance prior to HCT may limit the benefits of prebiotic fibres.

Future studies should consider long-term effects of fibre supplementation on clinical outcomes, including survival, recovery, and quality of life. Interventions should explore mechanisms by which different fibre types influence clinical outcomes and appropriate theoretical models. Large-scale RCTs with statistically powered samples and standardised controls should account for habitual dietary intake, degree of malnutrition, age, and antibiotic use as known confounders to microbial diversity. When publishing and conducting reviews, we encourage researchers to use the term prebiotic as a keyword and inclusion of additional terms, e.g., synbiotic, and specific fibre terms, such as resistant starch and fructooligosaccharides, as appropriate.

## Figures and Tables

**Table 1:** PICO template of inclusion criteria.

PICO	Inclusion Criteria
Patient	Adults 18 years and over with histological confirmation of haematological malignancy receiving haematopoietic stem cell transplantation.
Intervention	Oral prebiotic fibre dietary advice or supplementation.
Comparison	Usual care or no prebiotic fibre supplementation.
Outcomes	Feasibility measures, e.g., adherence and acceptability and recruitment. Clinical measures of gut health, immune function, gastrointestinal side effects (e.g., diarrhoea and constipation), quality of life, morbidity, and mortality. Mechanistic measures, e.g., microbiome (relative abundance and Shannon index).

**Table 2:** Quality assessment of the selected studies using the NOS. The details of the specific criteria the studies were assessed on can be viewed in Supplementary Materials S1.

First Author	Study Type	Selection (Maximum 4 *)	Compatibility (Maximum 2 *)	Outcome/Exposure (Maximum 3 *)	Overall Score (Maximum 9 *)
Andermann et al.	Prospective cohort study	3	1	3	7
Yoshifuji et al.	Prospective study	2	2	3	7
Iyama et al.	Non-RCT	4	1	3	8

* denotes the number of stars available for each quality assessment category.

## Supplementary Materials

The following supporting information can be downloaded at: https://www.mdpi.com/article/10.3390/nu17182973/s1. Supplementary S1a: Newcastle-Ottawa quality assessment scale (for cohort studies); Supplementary S1b: Newcastle-Ottawa quality assessment scale (for case control studies); Supplementary S2: Potential microbiome confounder reporting from studies included in the review; Supplementary S3a: Potential microbiome confounder reporting from studies of clinical interest not included in the review (see limitations); Supplementary S3b: Additional papers of clinical interest-summary of prebiotic fibre interventions and outcomes (see limitations); Supplementary S3c: Reported outcome measures within potentially clinically relevant studies not identified by this search (see limitations).
