# Supplementary material for: A Systematic Review Evaluating the Impact of Fibre Supplementation on Gut Health and Other Clinical Outcomes in Adults with Haematological Malignancies During Haematopoietic Stem Cell Transplantation
Source: Nutrients. 2025 Sep 16;17(18):2973. doi: 10.3390/nu17182973 (PMC12472375; doi:10.3390/nu17182973)
Supplement: Supplementary file 1 [file nutrients-17-02973-s001.zip › nutrients-3753946-supplementary.pdf]

Supplementary S1a:

Newcastle-Ottawa quality assessment scale (for cohort studies)

The * next to certain criteria indicate the ones where the study can be awarded a star should it meet that specific criteria.

Selection

1. **Representativeness of the Exposed Cohort**
   a) Truly representative of the average________________ (describe) in the community *
   b) Somewhat representative of the average ______________in the community *
   c) Selected subgroup of individuals, such as nurses or volunteers
   d) No information provided about how the cohort was derived

2. **Selection of the Non-Exposed Cohort**
   a) Drawn from the same community as the exposed cohort *
   b) Drawn from a different population source
   c) No information provided about how the non-exposed cohort was derived

3. **Exposure Ascertainment**
   a) Based on secure records (e.g., surgical records) *
   b) Determined through structured interviews *
   c) Collected via written self-report
   d) No description

4. **Demonstration That Outcome of Interest Was Absent at Start of Study**

    a) Yes *

    b) No

Comparability

Comparability of Cohorts Based on Study Design or Analysis

a) Study accounts for _______________ (specify the most important factor) *

b) Study additionally accounts for other relevant factors *(can be modified to indicate a specific control for a second important factor)

Outcome

1. **Outcome Assessment**

    a) Independent, blinded assessment *

    b) Record linkage *

    c) Self-reported outcomes

    d) No description

2. **Adequacy of Follow-Up Period**

    a) Yes (follow-up period sufficient for outcome of interest) *

    b) No

3. **Adequacy of Follow-Up Cohorts**

    a) Complete follow-up with all participants accounted for *

    b) Minimal loss to follow-up with low risk of bias (e.g., > __% follow-up or a description provided for those lost) *

    c) Significant loss to follow-up (e.g., < __% follow-up) with no explanation

    d) No statement

Supplementary S1b:

Newcastle-Ottawa quality assessment scale (for case control studies)

The * next to certain criteria indicate the ones where the study can be awarded a star should it meet that specific criteria.

Selection

1. **Is the case definition adequate?**
   a) Yes, independently validated *
   b) Yes, determined through methods such as record linkage or self-reports
   c) No description
2. **Representativeness of Cases**
   a) Cases are consecutive or clearly representative of the population *
   b) Cases show potential selection bias or representativeness is not stated
3. **Selection of Controls**
   a) Controls drawn from the community *
   b) Controls drawn from hospital settings
   c) No description
4. **Definition of Controls**
   a) Controls have no history of the disease (endpoint) *
   b) No description of control sources

Comparability

1. **Comparability of Cases and Controls Based on Design or Analysis**

    a) Study controls for________________ (specify the most important factor) *

    b) Study controls for any additional factor *

Exposure

1. **Ascertainment of Exposure**

    a) Secure records used (e.g.surgical records) *

    b) Structured interviews conducted with blinding to case/control status *

    c) Interviews conducted without blinding to case/control status

    d) Written self-reports or medical records only

    e) No description

2. **Consistency in Exposure Ascertainment for Cases and Controls**

    a) Yes *

    b) No

3. **Non-Response Rate**

    a) Same response rate for cases and controls *

    b) Non-respondents described

c) Different response rates and no designation

Supplementary S2. Potential microbiome confounder reporting from studies included in the review

Study (yr)	Locale	Age (yrs) Average$^{\$}$ Or (N,%*)		Sex (N,%*)		Ethnicity (N,%*)		Transplant conditioning chemotherapy		Local Antibiotic prophylaxis regimens (N,%*)	Local antifungal prophylaxis	Additional in treatment antibacterial or fungal delivery (N,%*)	Dietary intake Reporting
**Andermann et al., 2021** (I:15, C:16)	USA	I	61$^{\$}$	I	Female (7,47) Male (8,53)	I	White (8,53) Asian/Black (5,33) Other (2,13)		Fludarabine plus Melphalan (all)	Oral Ciprofloxacin D0 till cellular engraftment<br><br>Aciclovir + Fluconazole D+1	Trimetoprim-sulfamethoxazole for Pnemocystis jiroveci D+42	Yes, physician discretion at a participant level.	Not reported
		C	65$^{\$}$	C	Female (9, 56) Male (7, 44)	C	White (11,69) Asian/Black (3,19) Other (2, 13)					No-carapenem Beta-lactams (I: 12,80; C: 9,56) Carbapenems (I: 3,20; C: 5,31) IV Vancomycin (I: 9,60; C 6,28)	
**Yoshifuji et al., 2020** (I: 49, C:142)	Japan	I	<55yrs: 34, 69* ≥55: 15,31*	I	Female (12,24) Male (37,76)	I	Not reported	I	**MAC** (33,67) **RIC** (16,33)	Oral levofloxacine /tosuflocacin conditioning till neutrophil engraftment (all except ones already started other antibiotic)	Not reported	Antibiotics with relatively high anti-anaerobic activity (Conditioning to day 28) Yes: I: 36, 73; C: 116, 82 No: I: 13,27; C: 25, 18	Not reported
		C	<55yrs: 34, 69* ≥55: 15,31*	C	Female (50,35) Male (92,65)	C	Not reported	C	**MAC** (75,53) **RIC** (67,47)				
	Japan	I	52.5$^{\$}$ (21-62)	I	Female (50,35)	I	Not reported	I	**Intensive**	Not reported	Not reported	Not reported	Not reported

**Iyama et al., 2014**<br><br>(I:22, C:22)					Male<br>(92,65)				CY +TBI<br>(5,23)<br>BU +CY (2,9)<br>**RIC** (15,68)				
		C	47[$]<br>(19-62)	C	Female<br>(50,35)<br>Male<br>(92,65)	C	Not reported	C	**Intensive**<br>CY +TBI<br>(4,18)<br>BU +CY (2,9)<br>**RIC** (16,73)				

Table X. Reports study protocols and reporting and data collection of potential confounders to microbiome abundance and diversity within the studies. I – intervention, C - control; M- Male, F-Female; D1C (Day 1 of conditioning treatment) D0 – Day of cell infusion, D+ (No days post cell infusion). *% rounded to the nearest integer. MC- myeloablative conditioning, RIC – reduced intensity conditioning. CY -Cyclophosphamide, TBI – Total Body Irradiation, BU – Budesonide, RIC -reduced intensity conditioning. Flu- Fludarabine. Antibiotics with relatively high anti-anaerobic activity included Meropenem, Imipenem-cilastatin, and Piperacillin-tazobactam.

Supplementary Table S3a. Potential microbiome confounder reporting from studies of clinical interest not included in the review (see limitations).

Study (yr)	Locale	Group	Age (yrs) Average$^{\$}$ Or (N,%*)	Sex (N,%*)	Ethnicity (N,%*)	Transplant conditioning chemotherapy	Local Antibiotic prophylaxis regimens (N,%*)	Local antifungal prophylaxis	Additional in treatment antibacterial or fungal delivery (N,%*)	Dietary intake Reporting
**Anderman n et al., 2021** (I:15, C:16)	USA	I	61$^{\$}$	Female (7,47) Male (8,53)	White (8,53) Asian/Black (5,33) Other (2,13)	Fludarabine plus Melphalan (all)	Oral Ciprofloxacin D0 till cellular engraftment<br>Aciclovir + Fluconazole D+1	Trimetoprim-sulfamethoxazole for Pnemocystis jiroveci D+42	Yes, physician discretion at a participant level.	Not reported
		C	65$^{\$}$	Female (9, 56) Male (7, 44)	White (11,69) Asian/Black (3,19) Other (2, 13)				No-carapenem Beta-lactams (I: 12,80; C: 9,56) Carbapenems (I: 3,20; C: 5,31) IV Vancomycin (I: 9,60; C 6,28)	
**Yoshifuji et al., 2020** (I: 49, C:142)	Japan	I	<55yrs: 34, 69* ≥55: 15,31*	Female (12,24) Male (37,76)	Not reported	**MAC** (33,67) **RIC** (16,33)	Oral levofloxacine /tosuflocacin conditioning till neutrophil engraftment (all except ones already started other antibiotic)	Not reported	Antibiotics with relatively high anti-anaerobic activity (Conditioning to day 28) Yes: I: 36, 73; C: 116, 82 No: I: 13,27; C: 25, 18	Not reported
		C	<55yrs: 34, 69* ≥55: 15,31*	Female (50,35) Male (92,65)	Not reported	**MAC** (75,53) **RIC** (67,47)				

**Iyama et al., 2014**<br><br>(I:22, C:22)	Japan	I	52.5[$] (21-62)	I	Female (50,35) Male (92,65)	I	Not reported	I	**Intensive** CY +TBI (5,23) BU +CY (2,9) **RIC** (15,68)	Not reported	Not reported	Not reported	Not reported
		C	47[$] (19-62)	C	Female (50,35) Male (92,65)	C	Not reported	C	**Intensive** CY +TBI (4,18) BU +CY (2,9) **RIC** (16,73)				

Table X. Reports study protocols and reporting and data collection of potential confounders to microbiome abundance and diversity within the studies. I – intervention, C - control; M- Male, F-Female; D1C (Day 1 of conditioning treatment) D0 – Day of cell infusion, D+ (No days post cell infusion). *% rounded to the nearest integer. MC- myeloablative conditioning, RIC – reduced intensity conditioning. CY -Cyclophosphamide, TBI – Total Body Irradiation, BU – Budesonide, RIC -reduced intensity conditioning. Flu- Fludarabine. Antibiotics with relatively high anti-anaerobic activity included Meropenem, Imipenem-cilastatin, and Piperacillin-tazobactam.

Supplementary Table S3b. Additional papers of clinical interest- summary of prebiotic fibre interventions and outcomes (see limitations).

Author, Year, country	Study Design	Patient population & sample size	Intervention	Control	Outcomes measured	Results summary
**Yazdandoust et al, 2023 Iran**	Pilot RCT	Adults with HM (20-50yrs) Myeloablative conditioning Allogeneic HCT<br><br>I n =20<br>C: n=20	**Intervention:** Synbiotic (prebiotic (FOS) + probiotic)<br>**Daily dose:** Synbiotic capsule (Familact, Zist Takhmir, Iran) with 10^9 CFU 7 bacterial strains + fructo-oligosaccharide (FOS) 1 per day after lunch<br>**Start:** 21 days pre transplant (D-21)<br>**Stop:** Day of transplant (D0)	Standard care no synbiotic	**Primary:** aGvHD incidence & severity<br><br>**Secondary:** Regulatory T-cells (CD4, CD25, FoxP3, Tregs) D+7 and D+28, incidence of positive blood cultures and infections, severe diarrhoea, mucositis; overall survival, AEs (CTCAE v3.0)	Synbiotic intake before and during the conditioning regimen of allo-HSCT patients had a reduction in the incidence and severity of aGVHD through the induction of CD4 + CD25 + Foxp3+ regulatory T cells. Tregs in patients with aGVHD significantly lower at 7 and 28 days and 12 month survival rate was higher in symbiotic group.

**Riwes, 2023**	Single centre prospective feasibility study<br><br>With phase 2 pilot	Adults with HM Myeloablative conditioning Allogeneic HCT<br><br>Feasibility<br>I: n=10<br>C: n=15<br><br>Phase 2: I: 50 and C:10	**Intervention:** Single prebiotic (resistant starch, RS)<br>**Daily Dose:** 20g Resistant potato starch (Bob's Red Mill) daily for 3 days (D-10 to -8) increasing to twice a day (40g) from day -7 (Pre-transplant) until day +100 post-transplant<br><br>**Start:** 10 days pre-transplant<br>**Stop:** 100 days post-transplant	Feasibility: Historical Control<br><br>Phase 2: placebo (Digestible starch)	**Primary outcome:** Feasibility of delivery and RPS adherence<br><br>**Secondary outcomes:** AE (CTCAE v5.0), absolute concentration SCFA (Butyrate, acetate and propionate), microbiome: longitudinal alpha-diversity, volume butyrate producing and resistant starch degrading bacterium. Plasma metabolites	≥80% received ≥70% of scheduled doses with an average of 84% adherence. No ax toxicities or AE.<br>Trend to preservation of or increased alpha diversity with RPS. No diff in butyrate producers or RS degraders. Significant increase in intestinal butyrate levels in RPS group. Differences in metabolome at Nadir and engraftment.

Supplementary Table S3c. Reported outcome measures within potentially clinically relevant studies not identified by this search (see limitations).

**Author (yr)**	**Group**	**Reported Clinical Outcomes**										**Reported Microbiome / metabolome outcomes**		
		**Diarrhoea**			**Mucositis**		**Infections / Immunological markers**			**aGvHD**	**Disease**	**Microbiome / metabolome**		
		**Duration (Days)**	**Incidence (%)**	**Max. Grade**	**Duration (Days)**	**Max Grade**	**Days of Fever >38.5°C**	**Reported Infection**	**Immune cell markers**	**Incidence (%)**	**Survival Rate**	**SI**	**Butyrate bacterial abundance**	**Faecal Butyrate Levels**
**Yozdandoust et al 2023**	Synbiont (Familact)		No diff. Grade 2-3 10% Synbiont	No Grade 4		No diff in high grade mucositis 5% synbiont		No reported diff in bacteremia 10% symbiont	>% increase Treg @D+28 with Synbiont * 2.54 +/- 0.94 vs 1.73 +/- 0.95* (p<0.001)	Less aGvHD with Synbiont 10% vs 40% (0.067) and Grades III-IV with Synbiont*	> OS at 1yr with Synbiont 90 vs 75%			

	control		vs 10% control			vs 10% control		vs 15% control	Significantly lower Treg in pts with severe aGvHD at D+7 (p=0.027)and D+28 (0.007)	0% vs 25% (p=0.047)	(p=0.234)			
**Riwes et al 2023**	Resistant Starch												alpha diversity was preserved or increased with RS. Controls reduced alpha diversity at nadir increased at 3mths	Significant increase in intestinal butyrate with RS at baseline and was maintained (p<0.001)
	control													

This table shows the key outcomes reported by included papers, where there is a significant difference between studies P-values are reported and * assigned if p<0.05. Other reported differences are non-significant. aGvHD – acute Graft versus Host Disease, OS – Overall Survival, RS- resistant starch (Potato)
